# Book Notices

**Published:** 1899-09

**Authors:** 


### BOOK NOTICES.

The Twelve Tissue Remedies of Schüssler, comprising the theory, therapeutic application, materia medica, and a complete repertory of these remedies. Homœopathically and Biochemically considered, by Wm. Bœricke, M.D., and Willis A. Dewey, M. D. Fourth edition re-written and enlarged. Philadelphia: Bœricke & Tafel, 1899; price $2.50; by mail $2.73.

In all the history of the literary productions of the homœopathic school of medicine, nothing is more surprising to the homœopathist than the popularity of Schüssler's system of medicine. Constructed as it is with the apparent intention of abridging Homœopathy; of making prescribing by Homœopathy an easy task; of giving it a more *scientific* appearance by building upon it a speculative philosophy that is strikingly like the old school of medicine; its undoubted effect is to eclipse Homœopathy and to cause the latter to be abandoned as an effete and useless method of practice. This result is accomplished in nearly all minds which have not been seriously trained in the true principles of Homœopathy, and have not been enlightened as to the enormous clinical value of homœopathic methods of practice.

No book in the school has ever enjoyed so wide a circulation. No author has ever had so many ambitious editors. No work in the school has ever had such a variety of independent and unrelated editions. All these varied editions have been noticed in this journal as soon as produced.

Schüssler' latest edition translated by Prof. Tafel was brought out last year. Our opinion of it is recorded in The Homœopathic Physician for December, 1898, page 554.

Now comes the fourth edition of Bœricke & Dewey's rendering of it. A review of the third edition was given in The Homœopathic Physician for May, 1893, page 300. We do not think we can add anything to the criticism there recorded. This criticism was in the main favorable for the reason that the authors had subordinated Schüssler's theories and fancies to the solid principles of the pure homœpathic method. The *provings* of the drugs are given; the guiding symptoms and characteristics are conspicuous, and the whole treatment of these drugs is like that usual in the homœopathic materia medica; and then there is a good repertory. All these considerations remove the tissue remedies from the domain of Schüsslerism to that of legitimate Homœopathy. Of course the editors have been conscientious in recording Schüssler's ideas and teachings. But those who use the book have abundantly more to found a prescription upon than these teachings.

The plan of the book is as follows: Part I, an introduction in which Schüssler's ideas are elaborated. Part II, a materia medica in which Schüssler's speculative suggestions for each remedy are given, and in addition its actual positive materia medica and characteristics. Any one opening the book at this part would almost imagine he was looking at an ordinary homœopathic materia medica. Part III is an alphabetical arrangement of diseases with the indications for the remedies. Part IV is the repertory.

Thus we have a complete book which is far more useful than it would be if published just as Schüssler wrote it. This volume is the only complete exposition of Schüsslerism and the only practical working arrangement.

It does not prevent the reader from learning what Schüssler's teachings are, but it also does not veil Homœopathy.

Schüssler's philosophy is delusive notwithstanding the popularity of it, and will be found to be a failure in practice.

Homœopathy is amazingly successful when accurately applied.

This book supplies much that is wanting in Schüssler's teachings to enable the practioner to make successful prescriptions.

### Leaders in Homœpathic Therapeutics, by E. B. Nash, M. D. Philadelphia: Bœricke & Tafel, 1899; 380 pages; price $2.50; by mail, $2.63.

This excellent book was brought out at the beginning of this year. Its appearance is significant of the change that is going on in the opinions of the profession, and in consequence a new and better standard of medical practice more in consonance with the teachings of Hahnemann, is prevailing in the ranks of medical practitioners. Demand creates supply. In this instance the supply is admirable, and none who wish to avail themselves of the marvelous powers of homœopathic practice will have to turn away in disappointment from this work.

Dr. Nash, the author, is a well-known standard homœopathist. He has been in close touch with the veterans of the new school of medicine and he has imbibed from them an accurate knowledge of the wonderful system of Hahnemann. He confesses this in his introduction, where he says. "In my younger days I found much pleasure and profit in reading the writings of Hering, Dunham, Wells, Lippe, and others who have now ceased from their labors and gone to their well-earend rest. I have carefully tested their teachings, and now that my own hair begins to grow frosty, I desire to leave some testimonial to the truth of those teachings."

The remedies are not arranged in alphabetical order and in order to find any remedy we must refer to the index.

The very first remedy treated is Nux-vomica. Glancing at the statements made we find the following:

"After aromatics in food or as medicine, particularly ginger, pepper, etc., and after almost any kind of so-called hot medicines. Also will benefit persons who have been drugged by mixtures, bitters, herbs, and so-called vegetable pills, etc." Then the author remarks, "This is putting it in too wholesale a fashion. It would be true if said that Nux-vomica will *often* benefit such cases. The fact is that it will benefit those cases in which the use of such drugs, aromatics, pills, etc., has brought about a condition that simulates the symptoms produced in the provings of Nux-vomica, or in cases to which it is homœopathic and no others. Another fact is that these things often do produce such a condition and that is one reason why so many physicians are almost invariably prescribing Nux-vomica, the first thing, in cases coming from allopathic hands, without even examining the case. But it is unscientific. We have a law of cure, and there are cases in which the Nux-vomica condition is not present but another more similar remedy must be given."

No finer sample of close homœopathic reasoning, of clear comprehension of the homœopathic principle and of accurate application of it, could be adduced than the foregoing quotation from the first page. Experienced prescribers of our school will recognize its truth, and will have their admiration excited by it, and will need nothing more to commend it to their regard.

Turning to Bryonia we find the following: "When writing upon Pulsatilla we noticed the characteristic action of that remedy upon the mucous surfaces. Here Bryonia acts just as characteristically, but it is so different. With Bryonia it is excessive dryness or lack of secretion in them. It begins in the lips, which are parched, dry and cracked, and only ends with the rectum and stools which are hard and dry as if burnt. The same condition is undoubtedly present in the stomach which is evidenced by the excessive thirst; which can only be satisfied by large drafts of water; a little does not satisfy."

What a striking idea of Bryonia we get from the foregoing. It is admirable, and, what is more, the idea will remain in the mind. It will not be forgotten. Such an idea is of immense help in getting a simillimum for a case. It means *success* in the curing of the case.

All through the book such statements as the above occur, and they are genuine helps. Need more be said?

### Mineral Products of the United States, 1889 to 1898. United States Geological Survey, (Department of the Interior), Charles D. Walcott, Director. David T. Day, Chief of Division of Mineral Resources.

This chart 36 inches by 23 inches, gives detailed amounts of products of this country. This includes not only the metals found within our borders, but such products as coal, stone, petroleum, natural gas, clay, cement, mineral waters, phosphate rock, salt, gypsum, grindstones, precious stones, and other like substances.

Quantities and amounts in value are given in parallel columns and the grand totals for each year. There is a supplementary table showing the totals of all products for each year since 1880. These charts are very valuable for those who deal in statistics; for Congressmen and others dealing with tariff questions, and for all who are interested in the growth of this country.

THE CHRISTIAN LIFE: Vol. II, No. 50, July to September, 1899. Published by the National Purity Association, 79-81 Fifth Avenue, Chicago, Ill.

This is a small tract devoted to the subject of sexual continence and purity. The following are the principles of the National Purity Assocition:

The divine right of every child to be well born and welcomed into existence.

The improvement of the race through the observance of pre-natal laws and improved environments.

The right of children to be wisely, lovingly instructed in all that pertains to the right use of the sex nature.

The character of children should be improved by right thought and action on the part of parents during the pre-natal existence of their children.

Parents can, and should, endow their children with a better heredity than they themselves possess.

The destruction of the germ of life after conception is murder.

The only right means of limiting the number of offspring is continence and the exercise of wise self-control.

Procreative intercourse must be had only when offspring is mutually desired, wisely designed, and can receive the best inheritance the parents can give.

A fallen man is just as guilty as a fallen woman. "A white life for two," both in and out of marriage, is scientific, scriptural, and the only right way to live.

Marriage should be a sacred institution, where the sweetest, purest, holiest communion takes place, where "love is life and truth the light."

The true home is the most vital factor in the elevation of mankind and the prosperity and greatness of a nation, therefore, the purity and enlightenment of the home, through its individual members, transcends every other movement in importance and magnitude.

"Life force consecrated to highest use, is the divine law which man did not make, nor can he override it with impunity."

We believe in the right of woman to determine when she shall assume the maternal office.

The two things that most powerfully affect humanity for good or ill—heredity and environment—should be made as good as possible.

Ignorance is a fruitful source of vice; therefore wise instruction of the young is a good preventative, and a wise investment.

A child born of mutual love, wisdom and goodness, rightly environed, is sure to be a blessing to the world.

Stirpiculture—the improvement of humanity through pre-natal influences, etc.—is a most important science.

We believe that greatest good can come to humanity only through the observance of true and righteous rules of life, that exalt the soul, purify the mind and give strength and solidity to the moral nature.

Life may be and should be as pure and holy in its inception as it is possible for it ever to become.

Vital force wasted produces exhaustion and a demand for stimulants—resulting in widespread intemperance.

Vitality retained is transmuted into brain, nerve and mind force.

The Scriptures, rightly interpreted, favor the highest purity.

THE TREATMENT OF CAGE BIRDS. The most complete book of the kind ever published, irrespective of price. Mailed to any address on receipt of 15 cents by the Associated Fanciers, 400 N. Third Street, Philadelphia, Pa.

We all love birds, but few know how to care for them properly. Every one owning a bird will therefore be interested in a book containing over 150 engravings and a lithographic plate showing all the different kinds of fancy canaries in their natural colors; it gives full information in regard to song and fancy canaries and how to breed them for profit. Hints on the treatment and breeding of all kinds of cage birds, with description of their diseases and the remedies needed to cure them. All about parrots and how to teach them to talk. Instructions for building and stocking an aviary.

THE POULTRY DOCTOR. If you are interested in Poultry, by all means send 25 cents in postage stamps to the Associated Fanciers, 400 N. Third Street, Philadelphia, Pa., for their new book, on the diseases of poultry. Although comparatively small, it is so concise, terse and lucid as to be of great value to the fancier and the breeder of poultry. It shows how to manage and rear fowls, how to detect their different ailments, and how to treat them by either allopathic or homœopathic remedies. It is from the pen of Mr. John E. Diehl, the well-known American Poultry Association Judge, one of the highest authorities on poultry.

THE DOMESTIC CAT. The prominent attention lately bestowed upon the domestic cat by fashionable society and the great success of several cat shows have induced Mr. John E. Diehl, the well-known authority on domestic animals, to prepare a handy little volume under the above title. It carefully describes the different breeds and varieties, and states how to keep and rear cats; how to recognize their various diseases and how to treat them. The publishers' price for the book is 50 cents, but the Associated Fanciers, 400 N. Third Street, Philadelphia, Pa., will mail a copy of it on receipt of 25 cents to any subscriber of this paper.

